# Perceptions and Experiences of Consumer Representatives on Patient Safety Investigation Teams: A Qualitative Analysis

**DOI:** 10.1111/hex.70281

**Published:** 2025-04-30

**Authors:** Peter D. Hibbert, Yinghua Yu, Charlotte J. Molloy, Matthew Ames, Duncan Brown, Jenny Berrill, Zoe Fernance, Jennifer Morris, Liat Watson

**Affiliations:** ^1^ Australian Institute of Health Innovation, Faculty of Medicine, Health and Human Sciences Macquarie University Sydney New South Wales Australia; ^2^ IIMPACT in Health, Allied Health and Human Performance University of South Australia Adelaide South Australia Australia; ^3^ Independent Consumer Advisor Brisbane Queensland Australia; ^4^ Independent Consumer Advisor Sydney New South Wales Australia; ^5^ Independent Consumer Advisor Canberra Australian Capital Territory Australia; ^6^ Independent Consumer Advisor Melbourne Victoria Australia

**Keywords:** adverse event, consumer representative, patient safety, patient safety investigation

## Abstract

**Introduction:**

There is growing recognition of the importance of consumer representatives (CRs), consumers with lived experience, and advisors and volunteers in health systems to foster consumer‐oriented care. As part of this changing perception, some health services are inviting CRs to be on patient safety investigation teams. However, little is known from empirical studies about these representatives’ experiences and perceptions of their roles. This paper aims to contribute to understanding CR's involvement on investigation teams by examining the benefits, challenges, and other aspects of their participation.

**Methods:**

The study takes a qualitative approach and draws on data from interviews with 11 CRs and 10 focus groups comprising health service staff from Victoria, Australia, and a data interpretation workshop with an advisory panel of six consumers across four Australian states. Thematic analysis was used for data analysis.

**Results:**

We found that CRs have positive experiences in patient safety investigation teams, and their involvement often leads to more patient‐focused reviews and outcomes, and use of plain language. However, they also experienced some challenges, such as not being fully respected as equal members of the team, feeling uncomfortable speaking up, and practical issues such as payments and access to documents. The chair/facilitator plays a significant role in engaging with CRs meaningfully, and directly shapes the behaviours of the entire investigation team. Both CRs and chairs/facilitators require considerable institutional and systematic training and support within health services.

**Conclusion:**

The benefit of CRs on investigation teams outweighs the challenges across the individual, team/organisational and health systems levels. More resources and policies are needed to support CRs' sustainable inclusion, diversity and involvement in our current and future health services.

**Patient or Public Contribution:**

Eleven individuals with experience as CRs in patient safety investigation teams were interviewed. As indicated by the co‐authors of this paper, six consumers from four states who are members of a research consumer advisory committee have been involved in co‐designing the study methodology, interpreting the data, and writing this paper with the research team.

## 1 Introduction

### 1.1 Setting

Over the last 30 years, there has been a growing understanding that the health care system can, and does, harm patients (adverse events) [1]. Large scale studies, across many countries, have found that approximately 10% of hospital admissions are associated with an adverse event [2]. Examples of adverse events include incidents with medications (e.g., wrong dose or drug), infections (e.g., associated with surgery or intravenous catheters), or physical or mental health deterioration not being detected and managed in a timely way. Some adverse events can lead to serious or permanent physical disabilities and psychological trauma [3, 4].

When a serious adverse event occurs, health systems/services generally form a team to undertake a patient safety investigation or review to understand what happened and why, and to propose changes to reduce the risk of similar adverse events occurring in future [5]. Generally, the investigation teams are comprised of subject matter experts in relation to the clinical specialties involved in the adverse event (e.g., emergency department or surgery), health service safety and quality personnel, and potentially human factors experts [6]. The investigation or review may also inform the related but distinct process of “open disclosure”—disclosing information to the patient and family on why the adverse event occurred, and what changes the health service intends to make to prevent a similar incident from happening again [7]. The health care response, which includes the investigation and open disclosure, if not managed well, can compound the psychological harm to the involved patient and family. This can manifest in feelings of being inconsequential, powerless, manipulated, abandoned, dehumanised and disoriented [8].

One of the criticisms of patient safety investigations is they primarily focus on the clinical issues directly leading to the adverse event, and not enough consideration is on the whole patient journey, including the therapeutic relationships and communication between, and shared perceptions (or otherwise) of, clinicians and patient(s) [9, 10, 11]. There is growing interest in whether having a consumer representative (CR) on the investigation team may improve patient safety investigation teams' ability to develop a more balanced understanding of the adverse event and improve their focus on delivering outcomes that meets the needs of consumers, including open disclosure. Including a CR may foster dialogue that informs the understanding both of the adverse event, and proposed actions to prevent future occurrences [12]. However, given that investigations are inherently stressful, there may be a risk of psychological harm of inviting CRs onto investigation teams without appropriate support [13, 14].

In 2023, Safer Care Victoria implemented a consumer partnership framework that promotes including CRs as members of patient safety investigation teams [15]. The role of CRs is to: (1) be independent of the adverse event; (2) advocate for the patient to remain central to the review process; (3) challenge assumptions and identify gaps that may not be visible to staff; and (4) help address community expectations around public oversight and accountability. As of 2023, 89% of investigation teams in the Victorian public health system included a CR [3].

However, CRs' experiences and perceptions, including their engagement before, during and after the investigation, are rarely studied. This paper aims to fill this gap by exploring the benefits, challenges and other aspects of their involvement in investigation teams.

### 1.2 Research Gap

Consumers and consumer representatives have become key partners in many health systems internationally [16] and in Australia [17, 18]. Over the past decade, there has been growing recognition of the importance of CRs, consumer advisors [19] and volunteers in health systems to foster consumer‐centred care [20, 21]. However, little is known about these individuals' perceptions and experiences in empirical studies. Busch et al. (2021) conducted a literature review on active participation by patients in patient safety investigations and found only six papers on relevant topics from the perspectives of patients who were harmed. No papers related to CRs' involvement in patient safety investigations [22].

The literature on CRs in broader roles in health care may offer some insights for the more specific case of CRs on investigations. Wales et al. (2021) studied the experiences of CRs on various health care engagement activities in New South Wales, Australia, noting the importance of respect when acknowledging their time investment [23]. They identified respect as “being meaningfully included, supported, and heard, and activities needed to be purposeful and relevant.”^(p64)^ These principles could potentially be applied to the inclusion of CRs in investigations. Adams et al. (2023) point out that the role of CRs evolves over time, and health systems need to acknowledge their roles and motivations to be involved, and actively build a partnership with them, which will, in return, improve consumer participation [24]. Johnson et al. (2006) also observe that specific support needs to be developed to increase consumers’ effectiveness, capacity, and confidence to fulfil the representative role [25].

Much of the literature on CRs, is focussed on roles distinctly different from participation in a patient safety investigation. These roles often involve longer consumer‐health service relationships. On the contrary, an investigation is generally a short‐term assignment (usually under 3–4 months). Therefore, relationships and power dynamics between the CR and the rest of the team may be different to those that develop over time, for example, with a CR who is a member of a health care safety and quality or accreditation committee. An investigation is also focussed on a particular adverse event with, as noted above, an involved patient and family who may be suffering, and attempting to process, understand, and adjust to physical and psychological harm, including grief. Staff may also be suffering grief, guilt and shame. The investigation, and the overall health care service's response to an adverse event, potentially profoundly impact on many people's personal and professional lives over a long period. Therefore, the perspectives and experiences of the new role of CRs and their counterparts on patient safety investigations are important to understand.

### 1.3 Aims

This paper aims to examine the perceptions and experiences of CRs in relation to their roles on patient safety investigation teams.

## 2 Methods and Framework

### 2.1 Research Design

This study adopted a qualitative research approach, drawing data from focus groups with health services staff and semi‐structured interviews with CRs. The study was conducted in accordance with the Northern Sydney Local Health District (NSLHD) Human Research Ethics Committee (approval number: 2023/ETH02007). This study is part of a wider research programme on improving the health systems' response when patients are harmed [26]. The study is co‐authored by all six members of the overall research programme's consumer advisory committee, whose perceptions and insights were invaluable.

### 2.2 Setting

Health services in the state of Victoria, Australia.

### 2.3 Participants

Eleven CRs with experience participating in patient safety investigation teams (interviews), and 26 health service staff with experience working with CRs on investigation teams (in 10 focus groups).

### 2.4 Recruitment

Study participants were invited to participate by health service Directors of Clinical Governance/Safety and Quality, or Safer Care Victoria (SCV). The Directors or SCV identified a list of potential CRs and staff to participate and contacted them by phone or email. Most of the CRs were recruited through SCV. Those who expressed interest then received a formal email invitation, with a link to a RedCap participant information and consent form. RedCap is a secure web platform for building and managing Australian‐based online databases and surveys (https://redcap.mq.edu.au/). Following the completion of the consent form and demographic information, the research team contacted the participant to organise a time for the interview and/or focus group.

### 2.5 Data Collection

The research team conducted one‐to‐one interviews with CRs and focus groups with health services staff via Teams between April and July 2024. The main data source was interviews with CRs, designed as semi‐structured schedules (Supporting Information: Appendix A) that were developed from the patient safety literature [27], with a focus on CRs' perceptions and experiences of their roles, including benefits, challenges, risks and training and support required. Focus groups with health services staff were mainly centred on their experiences within investigations more broadly, with supplementary questions also asked around their experiences working with CRs on investigation teams—these were collected to provide a complementary perspective to the CRs.

### 2.6 Data Analysis and Interpretation

All the interviews and focus groups lasted 45 min to 1 h and were transcribed by Microsoft Teams and verified by YY. The transcripts were then uploaded to NVivo 14 for thematic analysis, employing an inductive reasoning approach. The research team then conducted a codesign workshop with a consumer advisory committee, where the interview results were shared and discussed.

We conducted three stages of data interpretation. First, one of the authors, (YY), read and reread the transcripts and independently coded them with themes emerging from interviews. The principal investigator, (PH), reviewed all the themes. YY and PH had several detailed discussions about the final selection of themes, refining them through their extensive knowledge of working in patient safety and sociology. Finally, in the codesign workshop, the research team asked the consumer advisory committee for their insights in interpreting the data.

## 3 Results

All 11 CRs came from various professional and cultural backgrounds and had diverse investigation experience, ranging from 1 to 20 years. Their average age was 58, with a range 35–71 years; seven were female (Table 1). They also have diverse experiences in investigations, ranging from leading investigation teams, participating in the entire process, to combinations of various activities such as initial meetings, timeline planning, patient interviews, and analysis meetings.

**Table 1:** Pseudonym names of consumer representatives who participated in interviews.

No.	Pseudonym	Gender
1	Margaret	Female
2	Wendy	Female
3	Francesca	Female
4	Philippa	Female
5	Fiona	Female
6	Heather	Female
7	Lyon	Male
8	Steve	Male
9	Pierre	Male
10	Rina	Female
11	Brett	Male

Ten focus groups were conducted with 26 health services staff in Victoria. The average age of participants was 49 years (range 36–68); 73% of participants were female. Eighteen of the 26 staff completed a demographic survey. Those 18 people primarily worked in nine different health services, with 89% primarily working in a metropolitan health service. About three‐quarters (72%) of people worked in primarily nonclinical roles, 92% of which were in Quality and Safety/Clinical Governance. Of the 28% working in a clinical role, 60% worked in general medical. All 18 staff had over 11 years' experience in the health system, with 72% having over 20 years' experience.

Five high‐level themes (outlined below) were developed from the data: the roles and benefits of CRs on investigation teams; challenges and risks of being a CR; the role of the chair or facilitator in the investigation; ideal training and support for CRs; and personal and professional characteristics of CR.

### 3.1 Theme 1: Role and Benefits of CRS on Investigation Teams

There was considerable overlap between our participants' discussions of the role and benefits of having a CR in the investigation; therefore, they have been combined. All 11 interview participants and staff members felt there were significant benefits to having at least one CR as a member of an investigation team. They have a common perception that having a CR supports the team to remain centred on patients' needs and experiences and bringing a consumer voice into the discussions.

The family have been given the opportunity to ask questions and they're there as part of the brief that we get. So one of the things I always do is I make sure we come back to those questions, and we pick them up. They don't just get missed.

(Steve)

There is a lot of medical terminology used so it's about sort of bringing that back to in a general sense, what does that mean to me as a patient?

(Philippa)

And they sometimes are excellent at asking tricky questions that to them they're just trying to clarify something but it's it helps you stop and thinking, oh, oh, actually, yeah, we didn't think about that.

(Health services staff focus group, VIC2)

One part of bringing the consumer voice to the investigation, may be preventing the team from blaming the patient.

Now that we've decided we don't blame individual practitioners, we blame patients an awful lot…I do a lot of work on like why are we blaming the patient… and is that valid?

(Margaret)

Some CRs also emphasised the importance of bringing their professional knowledge and skills into the investigation, which often assisted with navigating the power dynamics within the investigation team and disrupting inculcated professional and institutional culture. In doing so, CRs may assist in redressing imbalances in investigation team perspectives, by keeping some of the focus of the team on issues that the CR believe are important.

Of course, I asked questions. And every time, you know, I sort of spoke about… empowering patients to speak up as well when… their gut feel is like, oh, this isn't right.

(Francesca)

I guess being an engineer, we are able about the logic and rationality… it's a different perspective… I'm… no smarter than any of the clinicians that I come across. But you can just put across a view that's there that comes from a different direction.

(Brett)

The CRs also had a role in keeping the team focused on patient safety and an understanding of the impact that the report's finding may have on the patient and their family.

I think the role (CR) is to help keep the investigation patient‐centred I guess. A lot of the reviews and discussions gets very technical very quickly and I understand that… But ultimately, the report, the findings may need to go back to that to the patient's family or the patient themselves.

(Lyon)

Feedback from health services staff focus groups also suggested the immediate change observed with a CR in the investigation, which often improved the focus on consumer experiences and needs, opening up the investigation to different questions, and using plain language to communicate:

When they're [CRs] on a panel, I think of the two things that change immediately. One is people are much better at slowing down, explaining shorthand language, and thinking about it… from a consumer framework. And they sometimes are excellent at asking tricky questions, to them they're just trying to clarify something… it helps you stop and think… we didn't think about that….

(Health services staff focus group, VIC 2)

### 3.2 Theme 2: Challenges and Risks of Being a CR

#### 3.2.1 Challenges

While most CRs were optimistic about their involvement on investigation teams, there were challenges at multiple levels. A key challenge that three participants mentioned was a lack of respect for their value if their involvement is not fully understood at the investigation team level. This meant that CRs felt they must constantly prove the value of their participation in the investigation and their equal right to voice their opinions.

They don't think you should be there in the first place. They're the ones I work hard to convince that I am a valuable member of the team… it makes me quiet for a while… you have to sort of prove yourself nearly every time.

(Fiona)

This suggests that health services still need to provide greater emphasis and leadership to promote respect and recognition of CRs' value in patient safety investigations. It is also heavily influenced by the role of the chair/facilitator, who should ensure a consumer‐centred approach towards meetings and investigations.

I think it's very important that the person who's chairing the meeting just simply gives you (CR) the opportunity to speak at the appropriate time.

(Brett)

Health services staff also mentioned that it could be quite challenging for a CR to speak up in the panel discussions if the environment was not supportive, as they were often viewed as non‐experts.

… people [consumer representatives] actually need to be really supported and feel confident to be able to speak up in that space… It's helping them feel, but you are the expert in being a consumer much more than anyone else in the space… I'd have two of them on every panel ‘cause they could back one another, but I'd never be able to organise the panel meetings…

(Health services staff focus group, VIC2)

Similar statements were echoed by CRs.

…sometimes you feel a little bit unsure when you first walk in on a new panel… You have that right as the consumer, and you need to have a voice, and they need to invite you to speak if you haven't been given a voice.

(Fiona)

Two CR participants mentioned differentiated access to information and documents, which made it difficult for them to have a complete picture of the investigation.

…like some of the practical ones…access to documents…the bureaucracy I go through, and the health services panic… insert name of complicated system won't let us put you on unless you've got an institutional email address…

(Margaret)

CRs noted the financial challenges of appropriate and sustainable remuneration for CRs. A low level of remuneration disrespected CRs' engagement with the system. If not appropriately addressed, this issue would discourage CRs from further participation in the investigation.

There's the remuneration aspect. Most hospitals don't remunerate them [consumer representatives]… they expect that they just do that out of the goodness of their hearts. They wouldn't expect that of their health service staff… who are being paid by proxy of being around that table, but a consumer representative who can afford to do that…with the lack of remuneration comes only people who can afford to take time off, who are in a position to be able to do that.

(Wendy)

On the other hand, another CR felt that payment potentially reduced the CR's independent voice.

I don't want to be paid. I want to be able to speak about it. Otherwise, I don't see it as helping or supporting a patient… If you are not paid, then you are that free voice, an advocate and you can challenge from an outside point rather than inside…

(Rina)

One challenge identified was the CRs may not know the person or mechanism to escalate concerns with the investigation:

So I'm thinking of a couple of other instances with CRs … who have had a real concern about how a panel was operating and the lines that it was going down of the investigation and bringing them up to… the chair of that panel and sort of being pooh poohed or where do I go after that do I speak to the executive sponsor? I don't know who the executive sponsor is.

(Wendy)

### 3.3 Risks

Most participants felt the benefits of being a CR in the investigation strongly outweighed the risks. However, some still shared concerns about potential risks, which often reflected the outcome of discussions and impacts on individual CRs, for example, not being fully valued as a CR on the panel and exposure to traumatic events.

Other risks could be related to the locality of health services. For instance, small and rural communities may lack access to diverse perspectives, there may be privacy concerns, and there is a limited pool of CRs to draw on.

…in small places, there's the barrier of knowing somebody involved. So that is because in small places everybody you know knows somebody.

(Fiona)

Particularly in small communities, but applicable to all health services, is being mindful of cumulative and ongoing exposure to traumatic events.

… we've got a one public health service in this community, and we've got to be really mindful of the impact of exposing them to the understanding about what happens. We might include them in one [investigation] in a year but try not to involve them in other incident reviews and that sort of thing after I just sort of in terms of exposure to those traumatic events.

(Lyon)

On the other hand, one CR, Margaret, cautioned against making assumptions that CR could not cope with the stress of the investigations and the associated adverse events:

It's also very common… for the health services to put forward literally in writing their excuse for not involving consumers, saying oh it would be upsetting for the consumer. They (the health services) infantilise them, and they decide on their behalf that they'd be too distressed by being involved, when all the anecdotal evidence, which is all I've got to work with, is that consumers want to turn that power, you know, back around and actually have their opportunity to be heard. But they just write, we didn't involve consumers, they'd be too distressed, it's inappropriate, rather than letting the consumer make that choice for themselves.

(Margaret)

### 3.4 Theme 3: The Role of the Chair or Facilitator in the Investigation

Seven out of 11 participants mentioned the importance of the chair or facilitator in the investigation. The chair was crucial to influencing whether CRs would feel comfortable speaking up during an investigation. The chair also plays a significant role in pre‐meetings, preparing all the parties involved for the specific case, and the expectations of their roles in group discussions.

It's the standard thing in the facilitation of making sure that everybody is heard that no one person dominates the discussion, that everybody's point of view is brought in and is respected and well heard, and when there is conflict in the group, that the facilitator manages that in. There can be a range of ways of doing that, but they don't just either shut it down or pretend it's not happening.

(Steve)

So before we do an investigation like tomorrow, if it was, you know something quite big, we'd have a pre‐meeting with the chair and the consumer. I've gone through the case with them. They have an understanding, but you might have to go through some definitions of stuff…

(Health services staff focus group, VIC2)

### 3.5 Theme 4: Ideal Training and Support for CRs

Our data shows that CRs have different needs for support, varying from individual to organisational practices. Seven out of 11 participants emphasised the importance of having psychological and emotional support available, although only two mentioned having used such a service.

Nevertheless, they maintained it is essential to have access to such services and support if needed.

…having access to EAP's (Employee Assistance Programs) wherever health service they work at the same as the staff would have. Some people find that useful, some people don't. But I think that's not an unreasonable request that if you're doing work for health service that you should have access to any EAP program that they have access to…

(Margaret)

Other supports required included: a consumer representative community; ready access to relevant information and documents; further training on investigation methodology; the opportunity to observe clinical tasks (e.g., surgery, admission); and preparation of consumer representatives for the role and specific cases.

A commonly requested support is a community for CRs to be able to share their experiences:

…I would like to have it is a community practice in essence…we get together once every month just if you want to, and just talk about what it's like, who's figured out a great way to deal with someone who is being difficult in this way… practitioners get that. I don't have a union to join. I don't have a newsletter to read. I don't have conferences with my other consumer rep colleagues to go to… we miss out on all of that. To have anything approximating that I think would make a very big difference for a lot of us.

(Margaret)

While most participants participated in training before an investigation, there is a lack of consistency across various institutions. Participants generally found it helpful to receive training about systems thinking, investigation methodology and processes, and some basic training on medical terms. This helped them understand their roles and engage in panel discussions.

… there are short and long term, there's day seminars and things that people can do. I do think it's really helpful so that we know what the elements of the process are, and it allows you to not be baffled by how it works. I mean initially I do have to say on the first, I didn't have it before the first time I went on and that was a little more difficult dealing with the language, clinician talk.

(Rina)

Participants found understanding systems thinking was important:

Who have got the necessary training to understand just culture and systems thinking and like and why we don't want to just go in and you know sort of refer everyone to AHPRA (Australian Health Practitioner Regulation Agency) or you know, we don't want this blame culture. They've got to have some ability to understand that this is the ideal.

(Wendy)

CR Wendy believed that a national training programme needed to be established for those participating in incident investigations.

I think that you ensure that or that there is a national training programme for CRs that are going to be involved in incident investigations and be really clear with the industry what their roles are.

(Wendy)

The importance of having appropriate training for CRs was also echoed in the health services staff focus groups.

I think consumer reps are really valued, and I think they're really valued members of the review panel, provided they've got robust training.

(Health services staff focus group, VIC1)

### 3.6 Theme 5: Personal and Professional Characteristics of CRs

Empathy and the ability to speak up were the most frequently mentioned desirable characteristics of CRs.

I think it starts with empathy. You've gotta be able to put yourself in the position of clinicians… You also need people who've got empathy for the person who's been a sufferer”.

(Brett)

Necessary professional characteristics include knowledge about health systems, the investigation process, and ability to work constructively within a diverse clinical team.

Well, I think they need to be a people person for a start, so that you can work within a team.

(Heather)

I think also having some understanding of organisational culture and how that works…you do need to understand how people, how people think…

(Brett)

It's great when you do get a consumer who has got a, you know, a system focus from a different industry.

(Health services staff focus group, VIC1)

There is recognition of the need for diversity among CRs, including gender, race/ethnicity, language, age and culture, that reflect the broader community.

… are they always consumer reps we want to hear from? No, definitely not. If the issue involves somebody from multicultural community or a community… where they just think you know doctors are gods and must be you know, like agreed with and they are always right. Then you need to have a consumer representative that understands that culture and how that impacts and what we can do to mitigate that process in the future so that the families from that particular background are more empowered to speak up.

(Wendy)

There are also challenges of including CRs who are of working age. Eight out of 11 CR participants are at their retirement age and have more free time to participate in community work.

We need to encourage the younger ones to take part because you don't need old blood all the time telling new blood what to do. When I look at all those clinicians, they're so young now. This is a young system going on… so I think we need to make room for younger ones…mentor them so that they will come around and contribute.

(Rina)

Understanding the high workload involved in some investigations is important. Often, the incident investigation involves a lot of time‐consuming background reading before sitting at a panel discussion.

… because when you get 338 pages for a meeting of data. You know, dashboards and all of that, I'd think that's a lot of hours… consumers have to be aware that in the beginning that if you join particular groups, the reading may well be quite intense and especially if you're on adverse events because all those cases coming up need to be carefully read…. there's a lot of reading and involved. It's not just popping along.

(Rina)

Training and support of CRs was noted in the previous theme as important; health services have a responsibility to provide a level of training to ensure that CRs have the appropriate knowledge and skills to feel and be confident as a CR.

I think it's really important that consumers who are involved in panels have done the training. I think it was vital and if they were consumers that hadn't done the training, they'd be floundering. They'd be really floundering.

(Rina)

## 4 Discussion

This is the first study to investigate the underexplored area of CRs' perceptions and experiences in patient safety investigations. We found that both the CRs and their health service staff colleagues were positive about the role, in particular that the team was more likely to remain centred on the patient's and family's needs. The CRs may introduce questions or perspectives in the investigation that may not have been considered if they were not involved. Their participation may provoke changes such as more use of plain language.

Like other analyses of CR experiences in other roles in health services [23, 24, 28, 29], the presence of power imbalances between the CRs and other team members, and the need for respect for the CR role were cross‐cutting principles emerging from this study. We found the chair or facilitator of the investigation is crucial to dealing with conflict and imbalances of power [25], and to CRs being valued and heard. Our research aligns with other CR studies on the importance of leadership by the chair and of health services educating and advocating to staff about the importance of the CR role [24].

Tokenism is another theme in the broader literature related to power imbalance and respect [23, 30, 31, 32]. This may involve, for example, CRs being left out of key discussions, or unable to contribute ideas outside health providers' (or team's) priorities. Token involvement resulted in consumers feeling frustrated and dissatisfied [30]. Some CRs in our study found they had to ‘prove their value’ so their involvement was not tokenistic. Some had to do this repeatedly, as each investigation involved a different team. This emphasises the role of the chair in understanding the value of the CR and educating other members of investigation teams. This has health service‐wide implications—that modelling behaviours, leadership, support and commitment to ensure meaningful and valued CR involvement is embedded in all aspects of healthcare, not just investigations.

Similar to other studies [28], power imbalances and lack of respect can manifest in operational issues under the control of the health service, such as remuneration. Additionally, in our study, we found on some occasions, power imbalances were manifest, and respect was not afforded to the CRs, by the health service, by restricting access to documents. This made it difficult for CRs to fully understand and interrogate the adverse events they were supposed to be investigating.

At the heart of each investigation is a patient and family suffering harm. One of the important points made by many of the CRs in our study was describing their role as not to directly represent these patients or families but to present the, or indeed a, consumer voice. By doing this, the CRs may improve the person‐centredness of health services, and focus on patient's needs and experiences, without fulfilling the unrealistic and burdensome task of directly representing the patients and families involved in the adverse event. Ideally, they can present a consumer voice by being informed by their own personal experiences, professional skills and knowledge, and insights into the realities of the health system. For example, understanding transitions of care into the community and barriers to access care.

Patient safety investigations deal with difficult topics and can challenge relationships between people, departments and organisational structures. They deal with highly sensitive issues, are stressful, and can be emotionally confronting for all team members. However, participants in our study implored that health services should not default to infantilisation of CRs, by automatically assuming CRs will not cope, or indeed use the inherent stress of an investigation as an excuse to minimise CR involvement. Like broader CR roles, there is a need for appropriate supports, psychological and emotional and other (e.g., access to a community, training, preparation), and systems for recognising and responding to stress and distress. But participant CRs, all with experience participating in investigations, were firm in their resolve that they accepted the challenges this involved, such as contributing to a health service response that supports restorative justice for patients, families and staff involved, and to improve the health system.

Our research also shows we need to acknowledge the challenges of diversity and sustainability within CR communities, and the need to engage with people from culturally and linguistically diverse (CALD) backgrounds, and younger people who are of working age. This is echoed by research done by Chuachan [33] and his team [34, 35] on consumer engagement with CALD communities, and the complexities of peoples' experiences in this area. Such considerations would broaden our engagement with CALD consumer needs [36] and contribute to the patient safety culture and investigations [37].

### 4.1 Strengths and Limitations

To the authors' knowledge, our paper is the first to investigate the perceptions and experiences of CRs on investigation teams. A strength is that we also included perspectives from health service staff, which provided comprehensive and complementary perspectives to consolidate CRs' experiences. While our participants have a wide range of professional and personal experience, most have a Western and educated background. This was a consequence in part of the recruitment process but may also reflect wider patterns in who currently occupies CR roles.

Another strength of our study is that it engaged with CRs and an advisory committee. Therefore, our interpretation of the data is rooted in the practical experiences of consumers and CRs.

The primary data collection was conducted in one state in Australia; the experiences of CRs in other jurisdictions with other systems of investigations may not be generalisable.

## 5 Conclusion

Our findings highlight that CRs' involvement in patient safety investigations is multidimensional and complex. Firstly, the benefit of their engagement on investigation teams outweighs the challenges across the individual, team/organisational and health systems levels. In particular, CRs focus investigative processes and discussions on patient safety and needs of patients. Second, the role of chair/facilitator is crucial to manage power imbalances and fully and respectfully engage CRs on investigation teams. Their respect for CRs shapes the entire team's behaviour, and therefore the experience of CRs in that team. Third, unpacking CRs' perceptions and experiences of sitting on investigation teams helps us ensure their voices are respected, heard, and valued, and that their engagement with the broader health services is better informed, planned and delivered. More resources and policies are needed to support CRs' sustainable inclusion, diversity, and involvement in our current and future health services system.

Methodologically, we not only draw on qualitative data, but also maximise consumers' engagement and perspectives in our project through a collaborative and participatory approach.

## Author Contributions

**Peter D Hibbert:** conceptualisation, investigation, funding acquisition, methodology, validation, writing – review and editing, formal analysis, supervision, visualisation. **Yinghua Yu:** data curation, investigation, methodology, writing – original draft, formal analysis. **Charlotte J Molloy:** writing – review and editing, investigation, methodology, project administration, data curation. **Matthew Ames:** writing – review and editing, validation, methodology. **Duncan Brown:** writing – review and editing, validation, methodology. **Jenny Berrill:** methodology, validation, writing – review and editing. **Zoe Fernance:** methodology, writing – review and editing, validation. **Jennifer Morris:** methodology, validation, writing – review and editing. **Liat Watson:** methodology, writing – review and editing; validation.

## Ethics Statement

The study was conducted in accordance with the Northern Sydney Local Health District (NSLHD) Human Research Ethics Committee (approval number: 2023/ETH02007).

## Consent

All patients were informed that participation was voluntary and provided written informed consent before interview.

## Conflicts of Interest

The authors declare no conflicts of interest.

## Supporting information

Appendix A ‐ semi‐structured questions for consumers.

## Data Availability

All the data is confidential due to ethical considerations.
